# Enhancing Child Sexual Abuse Prevention Knowledge With an Educational Toolkit -Evaluation of the Chinese Doll Program

**DOI:** 10.3389/ijph.2024.1606641

**Published:** 2024-01-22

**Authors:** Ketong Xu, Jiuqiang Fu, Jianming Yang

**Affiliations:** ^1^ Tangshan Research Institute, Beijing Institute of Technology, Tangshan, China; ^2^ School of Design and Arts, Beijing Institute of Technology, Beijing, China

**Keywords:** child sexual abuse, CKAQ-RIII, intervention, evaluation, prevention

## Abstract

**Objective:** In countries with conservative attitudes towards sex and limited resources to prevent child sexual abuse, culturally adapted CSA prevention programs are essential. This study outlines a randomized controlled trial evaluating the effectiveness of the Doll program for preventing CSA in the specific context of China.

**Method:** 181 children were pre-tested and post-tested (5 weeks later) for knowledge of sexual abuse prevention. Children were assigned to one of three groups; 1) child only (*n* = 60); 2) child and parent (*n* = 60); and 3) control (*n* = 61).

**Results:** Children in groups 1 and 2 showed significant increases (*p* < 0.001) in scores on the Appropriate Touch Scale (ATS) and the Inappropriate Touch Scale (ITS), whereas those in the control group did not show a significant increase in ATS scores, but their scores on ITS significantly increased (*p* < 0.001). Children in group 2 showed significantly increased ITS scores compared to group 1 (*p* = 0.016).

**Conclusion:** Doll program effectively enhances children’s CSA prevention knowledge, with parental engagement demonstrating a positive impact on the program.

## Introduction

### CSA Prevalence and Prevention in China

Despite the rights of children to be safeguarded against all forms of violence, including sexual abuse, Child sexual abuse (CSA) remains a grave public health issue affecting children across diverse cultures and societies, with severe negative consequences for victims, their families, the social environment, and society as a whole [1–4]. Cultural norms related to parenting, discipline, family dynamics, and sexuality may influence the prevalence, forms, definitions, characteristics, and impact of child sexual abuse in different regions [5, 6].

China, being a hierarchical society with strong Confucian ideology, exhibits certain cultural aspects that may contribute to child sexual abuse [7]. For instance, the moral code of filial piety expects children to unquestioningly obey their parents, even if they are subjected to scolding or physical punishment, which is viewed as an expression of care [6]. The traditional Chinese cultural suppression of sexual topics makes it challenging for Chinese children to discuss sexuality and express experiences of sexual victimization [8]. Moreover, Chinese families often discourage CSA victims from disclosing their experiences to avoid bringing shame to the family [6].

With the rise of Internet technology, child sexual abuse is gaining more visibility in China. Shifts social values have led to increased awareness of “child abuse,” with the Chinese term “Nue Tong” being used to define child victimization [7]. Although several studies have shown the benefits of formal, systematic, and repeated training through school-based CSA prevention programs [9–17], these school-based CSA prevention programs, which incorporate educational materials such as courses and dramatic performances may not be fully applicable in China because of the lack of systematic education faculty [18], and their preparation and implementation may be impractical due to time and resource constraints in schools [19, 20]. Particularly during the COVID-19 pandemic, school-based interventions became unfeasible, emphasizing the importance of remote digital interventions as promising solutions for addressing resource limitations and expanding reach [21–23]. Additionally, discussing sexuality in public remains sensitive in China, making it challenging to implement abstract CSA prevention concepts that young children may struggle to comprehend. Moreover, a variety of digital resources for child sexual abuse (CSA) prevention education have been created to enhance program suitability for different age groups, increase engagement, and improve accessibility [16], [24–29].

### Description of the Doll Program

The Doll program is based on the aforementioned context of sexuality education in China and is specifically tailored for lower elementary school students. The Doll program is designed to address the challenges of limited educational resources, particularly the absence of sex education curricula in schools and the broader family environment. Therefore, the e-educational toolkit consisting of digital textual materials and engaging simple serious games as educational materials was developed for Doll program. While in the e-education toolkit, we use a lot of doll images to represent a lot of different characters in real life, such as strangers, doctors, parents, etc., so the name of the program is Doll.

**FIGURE 1:**
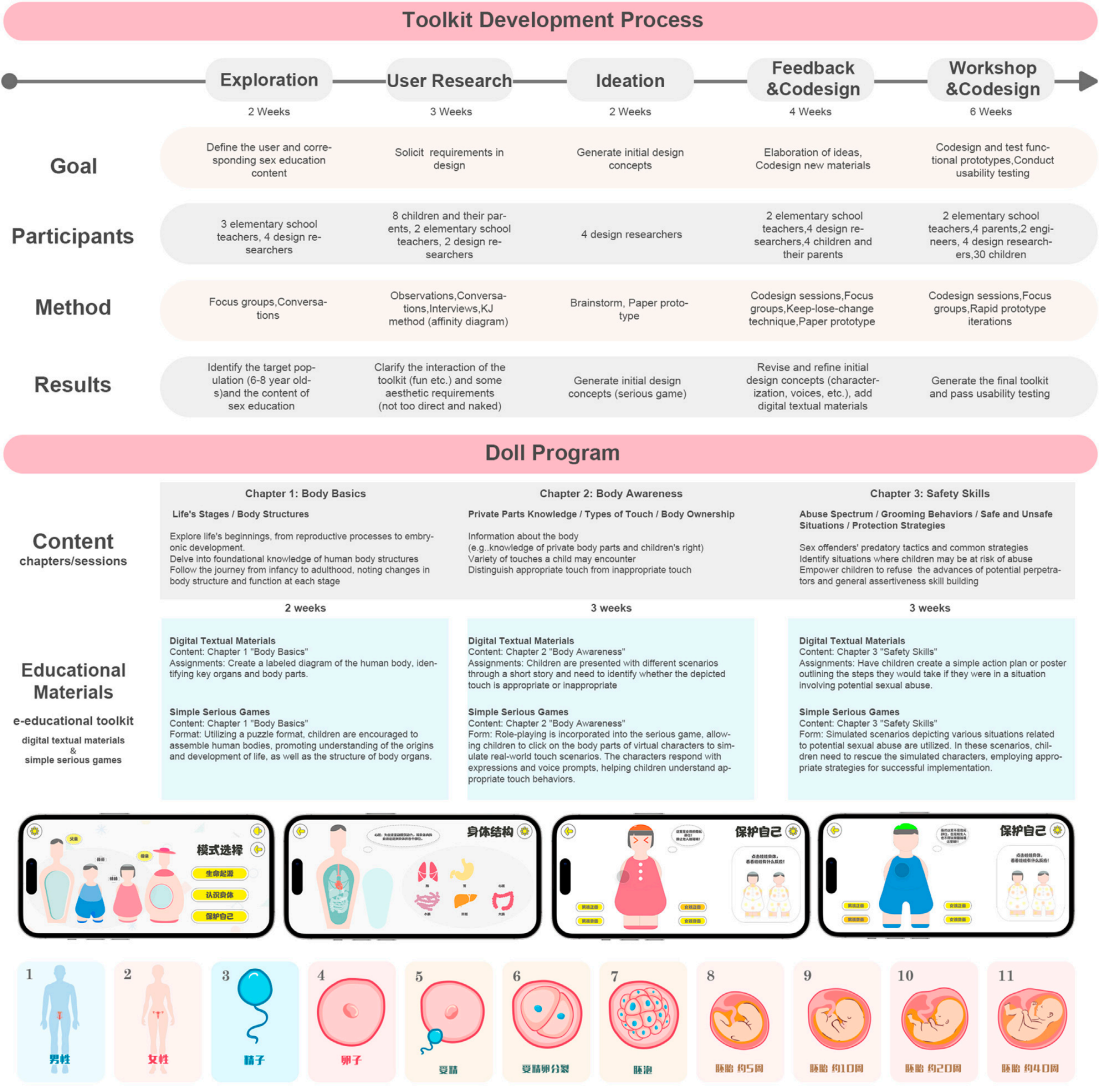
Description of the Doll program and the toolkit (China, 2022).

The content of CSA prevention in the Doll program is anchored in UNESCO’s 2018 International Guidelines for Sexuality Education (Revised) [30] and Chinese textbooks dedicated to elementary school children’s sexuality education—specifically, the 12-volume Cherishing Life series edited by Wenli Liu [31, 32]. Illustrated in Figure 1, the content is organized into three main chapters: body basics, body awareness, and safety skills.

The content of the e-educational toolkit aligns with the three aforementioned chapters. The development the toolkit (Figure 1) employed a co-design approach [33–36], involving various stakeholders, including parents, teachers, designers, and engineers. The e-text materials in the toolkit comprehensively cover the knowledge to be acquired in each chapter, accompanied by corresponding assignments. The toolkit’s games aim to enhance children’s understanding of abstract concepts through images, thereby fostering interest in learning. These games take the form of jigsaw puzzles, role-playing, and simulation situations. Given its specific design for Chinese children in the early elementary grades, the toolkit currently supports Chinese characters and Pinyin languages.

In our quest to assess the efficacy of the Doll program, we delve into the fundamental question: Does students’ knowledge of sexual abuse prevention experience a meaningful shift from pre to post-test? Drawing from a wealth of previous research highlighting the positive impact of parental involvement in CSA prevention programs [11], [37–39], we extended an invitation to children’s parents to actively participate in the evaluation. Thus, we have formulated the following hypotheses:

Hypothesis 1: Active participation in the Doll program will result in a significant improvement in students’ understanding of child sexual abuse compared to their counterparts who did not partake in the program.

Hypothesis 2: Within the Doll program participants, we anticipate that the child and parent group will demonstrate a notably more pronounced enhancement in child sexual abuse knowledge compared to the child-only group.

## Methods

### Participants

In the initial phase, we assessed the eligibility of 260 children with a Chinese cultural background from a prominent public elementary school in Beijing, China. Insights into their physical and cognitive status were gathered through information provided by their class teachers. Inclusion criteria required that children could understand and use the toolkit normally. However, five children exhibiting severe motor impairments, such as broken bones and an inability to write at the time of assessment, were excluded from the Doll program. Consent was obtained from both the children and their caregivers through the signing of an informed consent form. Unfortunately, 74 caregivers did not agree to participate, leading to the subsequent exclusion of 79 children. Ultimately, 181 children actively participated in the Doll evaluation. Among the participants, 49.2% were females (*N* = 89), and 50.8% were males (*N* = 92). The participants had an average age of 6.12, with a standard deviation of 0.78.

### Measures

The Children’s Knowledge of Abuse Questionnaire (CKAQ-RIII), developed by researchers based on previous studies, was utilized in this study to assess sexual abuse prevention knowledge among children [40–43]. The CKAQ-RIII comprises 33 items, with 9 items constituting the Appropriate Touch Scale (ATS) and 24 items forming the Inappropriate Touch Scale (ITS). ATS addresses concepts like appropriate touch, such as a doctor needing to examine private parts or seeking help from an unfamiliar security guard. ITS focuses on encompassing different touch types and encouraging children to communicate discomfort. The reliability of ITS and ATS within the CKAQ-RIII scale has been demonstrated in studies using either partial or full versions, including non-English speaking samples [16, 17, 41], [44–49]. This substantiates the CKAQ-RIII as a robust tool for evaluating children’s knowledge of sexual abuse prevention, supporting its application in both research and practical contexts.

The Chinese version of CKAQ-RIII has been utilized in previous studies [11, 50]. In the Chinese version of the questionnaire, “True,” “False,” or “I don’t know” was replaced with “Agree,” “Disagree,” and “Don’t know” as response options. A score of 1 was assigned to correct responses, whereas incorrect, “Don’t know,” or blank answers received a score of 0. In our study, the ITS (24 items) demonstrated strong internal consistency (*α* = 0.791), while the ATS (9 items) exhibited slightly lower reliability (*α* = 0.712). This level of reliability is considered acceptable and aligns with findings in other studies [11, 46, 50].

### Procedure

We obtained parental consent for all participating children, and the study protocol was approved by the ethical Institutional Review Board (IRB) at Beijing Institute of Technology.

A total of 181 children from 5 classes participated in the program and were randomly assigned to groups 1, 2, or 3. Group 3 served as the control group and did not participate in the Doll program (control group, *n* = 61). Group 1 used the toolkit alone (child only group, *n* = 60) in the Doll program. Group 2 used the toolkit with their parents (child and parent group, *n* = 60) in the Doll program.

Parents were briefed on research procedures, measures used, and participant rights during parental meetings. The Doll program was offered as an extracurricular school activity for group 1 children. Group 1 children completed the toolkit in the school’s multimedia classroom, supervised by two trained teachers. Group 1 teachers ensured that students used the toolkit individually. Group 2 children, using devices such as cell phones, tablets, or computers, completed the toolkit at home as part of their regular homework assignments with their parents. Parents actively engaged in their children’s toolkit use, guiding them, and participating in discussions.

Quizzes or tasks were implemented at the conclusion of each chapter of the e-toolkit to ensure children’s active involvement in learning. Additionally, parents in group 2 were provided with a paper version of the family learning log. They were asked to record their progress after finishing each chapter, ensuring active involvement in shared learning.

The CKAQ-RIII (Chinese version) was assessed twice during the summer trimester of the 2022 school year: 1) at time 1, to gauge children’s understanding of CSA concepts before the intervention, and 2) at time 2, around 5 weeks later following their involvement in the Doll program. In all cases, the administrator verbally repeated each question twice, giving every child sufficient time to respond or seek clarification. Overall, the children exhibited a strong understanding of the questions, and the need for repetition was minimal.

**FIGURE 2:**
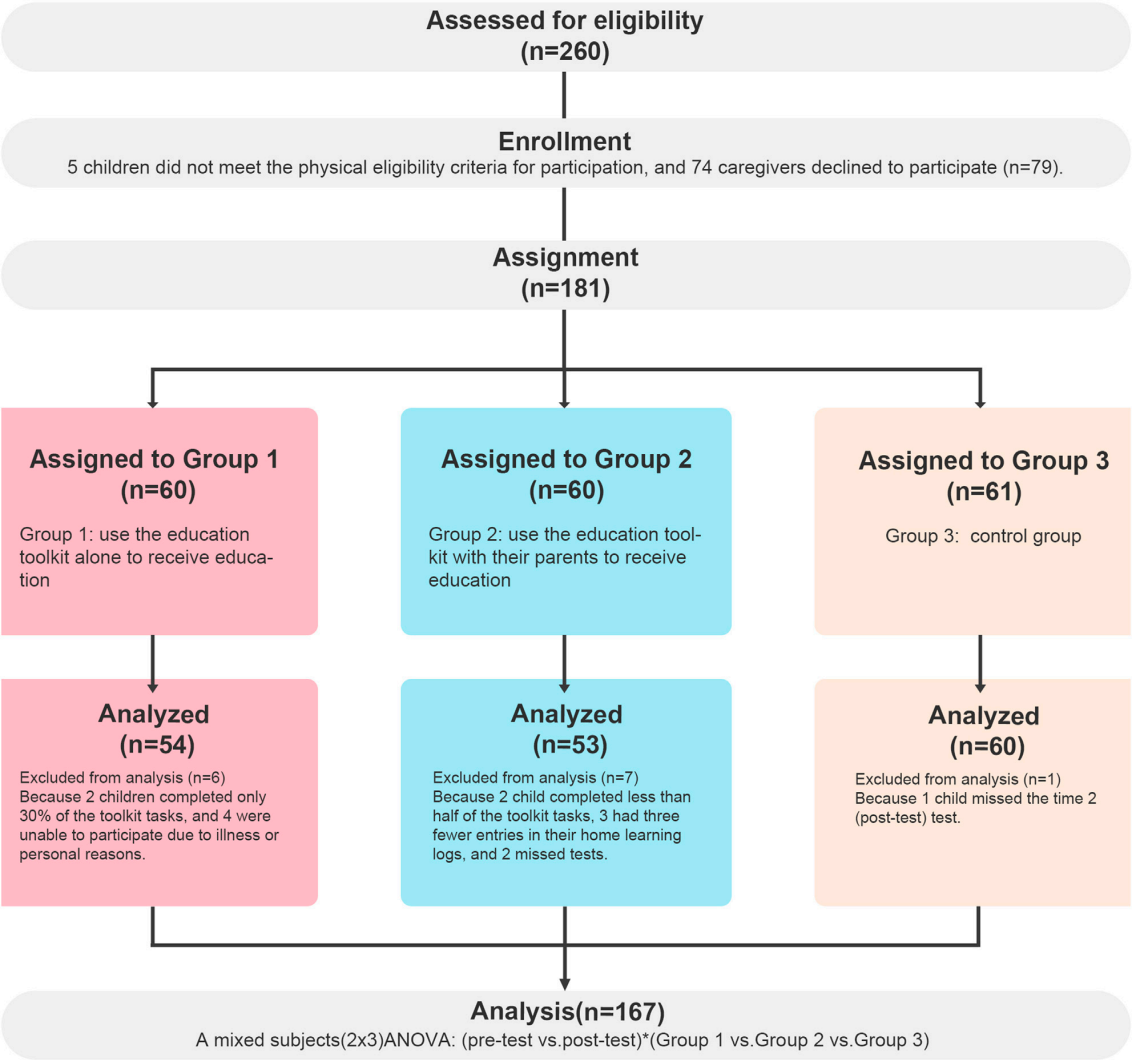
Sampling procedure of the study participants (China, 2022).

Students in groups 1 and 2 who did not complete at least 50 percent of the tasks or quizzes in the toolkit, as well as students in group 2 with predominantly blank home learning logs (fewer than three entries), and those who missed the tests due to illness, were excluded from the analysis. The final evaluation (Figure 2) included a total of 167 students: group 1 (child only), *n* = 54; group 2 (child and parent), *n* = 53; group 3 (control), *n* = 60.

### Data Analysis

We conducted a comprehensive analysis using Two-Way Mixed ANOVA to examine the interaction, main, and simple effects of the three groups on the two scales at two time points (Table 1). Subsequently, we performed separate multiple comparisons for the three groups’ scores on the two scales at each time point, employing the Bonferroni correction method (Table 2).

**TABLE 1:** Main, interaction effects, and simple effects analysis (China, 2022).

Measures	Variables		Pre-test	Post-test	Time simple effects			Main and interaction effects		
			M ± SD	M ± SD	df	F	Partial η^2^	df	F	Partial η^2^
ATS^a^(0–9)	Group 1 (*n* = 54)		4.33 ± 2.40	4.98 ± 2.46	1	12.550**	0.071			
	Group 2 (*n* = 53)		4.36 ± 2.50	5.13 ± 2.27	1	17.546***	0.097			
	Group 3 (*n* = 60)		4.27 ± 2.16	4.22 ± 1.95	1	28.840	0.001			
	Group simple effects	df	2	2						
		F	0.023	2.792						
		Partial η^2^	0.000	0.033						
	Time							1	19.258***	0.105
		Group							2	0.868	0.010
		Time x Group							2	6.263**	0.071
ITS^b^(0–24)	Group 1 (*n* = 54)		10.52 ± 3.69	13.33 ± 3.62	1	520.418***	0.760			
	Group 2 (*n* = 53)		11.17 ± 3.16	15.25 ± 3.32	1	1070.755***	0.867			
	Group 3 (*n* = 60)		10.63 ± 3.71	11.12 ± 3.54	1	17.019***	0.094			
	Group simple effects	df	2	2						
		F	0.519	19.726***						
		Partial η^2^	0.006	0.194						
	Time							1	1223.487***	0.882
		Group							2	6.294**	0.071
		Time × Group							2	230.136***	0.737

** indicates *p*-value < 0.01, *** indicates *p*-value < 0.001.

^a^ The CKAQ-RIII (Children’s Knowledge of Abuse Questionnaire) consists of 33 items, with 9 items forming the Appropriate Touch Scale (ATS).

^b^ The CKAQ-RIII (Children’s Knowledge of Abuse Questionnaire) consists of 33 items, with 24 items constituting the Inappropriate Touch Scale (ITS).

**TABLE 2:** Pairwise comparisons of inappropriate and appropriate touch scores among three groups at two time points adjusted by Bonferroni (China, 2022).

Measures	Time		Group 1	Group 2	Group 3
ATS^a^(0–9)	Pre-test	Group 1	—		
		Group 2	*p* = 1.000	—	
		Group 3	*p* = 1.000	*p* = 1.000	—
	Post-test	Group 1	—		
		Group 2	*p* = 1.000	—	
		Group 3	*p* = 0.207	*p* = 0.092	—
ITS^b^(0–24)	Pre-test	Group 1	—		
		Group 2	*p* = 1.000	—	
		Group 3	*p* = 1.000	*p* = 1.000	—
	Post-test	Group 1	—		
		Group 2	*p* = 0.016	—	
		Group 3	*p* = 0.003	*p* < 0.001	—

^a^ The CKAQ-RIII (Children’s Knowledge of Abuse Questionnaire) consists of 33 items, with 9 items forming the Appropriate Touch Scale (ATS).

^b^ The CKAQ-RIII (Children’s Knowledge of Abuse Questionnaire) consists of 33 items, with 24 items constituting the Inappropriate Touch Scale (ITS).

## Results

Our data adhered to normal distribution assumptions (Shapiro-Wilk’s test, *p* > 0.05) and demonstrated homogeneity in both variances (Levene’s test, *p* > 0.05) and covariances (Box’s M test, *p* > 0.001). The following sections present detailed results of this data analysis for each scale.

### ATS: Appropriate Touch Scale

ATS scores showed a significant interaction involving group and time [F (2, 164) = 6.263, *p* = 0.002, partial η^2^ = 0.071]. The effect of time was significant, indicating differences in ATS scores over time [F (1, 164) = 19.258, *p* < 0.001, partial η^2^ = 0.105]. Conversely, the influence of different groups did not uncover substantial distinctions in mean ATS scores [F (2, 164) = 0.868, *p* = 0.422, partial η^2^ = 0.010].

With regard to the simple effects of group (Table 1), there were no significant differences in children’s knowledge observed at time 1 (pre-test), F (2, 164) = 0.023, *p* = 0.977, partial η^2^ = 0.000. However, there was still no statistically significantly difference in all 3 groups at time 2 after the intervention, F (2, 164) = 2.792, *p* = 0.064, partial η^2^ = 0.033.

Regarding the simple effects of time (Table 1), ATS scores for students in group 1 (child only) exhibited a noteworthy enhancement from time 1 to time 2, F (1, 53) = 12.550, *p* = 0.001, partial η^2^ = 0.071. Similarly, ATS scores significantly improved for students in group 2 (child and parent) between the initial assessment to the follow-up evaluation, F (1, 52) = 17.546, *p* < 0.001, partial η^2^ = 0.097. Nevertheless, no statistically significant disparity emerged in ATS scores for students in group 3 (control group) from the baseline evaluation to the follow-up assessment, F (1, 59) = 28.840, *p* = 0.774, partial η^2^ = 0.001.

### ITS: Inappropriate Touch Scale

An evident interaction between groups and time was found for ITS [F (2, 164) = 230.136, *p* < 0.001, partial η^2^ = 0.737]. There was a clear difference in mean ITS score across the various time intervals, F (1, 164) = 1223.487, *p* < 0.001, partial η^2^ = 0.882. Notably, group membership also exhibited a significant impact on mean ITS scores, F (2, 164) = 6.294, *p* = 0.002, partial η^2^ = 0.071.

Regarding the simple effects of group (Table 1), children’s knowledge did not show a significant difference at time 1 (pre-test), F (2, 164) = 0.519, *p* = 0.596, partial η^2^ = 0.006. However, after the intervention, there was a significant difference at time 2 (post-test), F (2, 164) = 19.726, *p* < 0.001, partial η^2^ = 0.194.

Multiple comparisons (Table 2) revealed that group 1 demonstrated a notably higher ITS score (*p* = 0.003) in contrast to the control group (group 3). Likewise, group 2 exhibited a substantial increase in the ITS score (*p* < 0.001) compared to group 3. Additionally, there was a significant distinction in the ITS score between group 1 and group 2 (*p* = 0.016).

With regard to the simple effects of time (Table 1), in the child-only group (group 1), ITS scores notably increased from the initial assessment to the subsequent evaluation, F (1, 53) = 520.418, *p* < 0.001, partial η^2^ = 0.760. Similarly, the child and parent group (group 2) demonstrated significant enhancements in their ITS scores from the initial assessment to the subsequent evaluation, F (1, 52) = 1070.755, *p* < 0.001, partial η^2^ = 0.867. Although there was no intervention in group 3, ITS scores in group 3 significantly improved from the initial assessment to post-test (M = 11.117, SE = 0.452), F (1, 59) = 17.049, *p* < 0.001, partial η^2^ = 0.094.

## Discussion

The purpose of this study was to investigate the efficacy of the Doll Program in enhancing knowledge of sexual abuse prevention among early-grade elementary school children. We recruited 181 students from Beijing elementary schools, randomly assigning them to one of three groups: 1) child only, 2) child and parent, or 3) control group. The Children’s Knowledge of Abuse Questionnaire was utilized to examine pre-test and post-test scores on the two subscales—Appropriate Touch Scale and Inappropriate Touch Scale. The following outcomes are revealed in the data discussed below in relation to the hypothesized questions.

Addressing Hypothesis 1, our results indicate that involvement in the Doll program substantially improved awareness of child sexual abuse prevention, particularly in recognizing inappropriate touching. This signifies a notable advancement in child-specific knowledge.

All three groups exhibited similar levels of knowledge regarding appropriate and inappropriate touch before any intervention. The scores on the Appropriate Touch Subscale at pre-test (*p* = 0.977) and the Inappropriate Touch Subscale (*p* = 0.596) showed no significant differences among the three groups.

Both group 1 (*p* = 0.001) and group 2 (*p* < 0.001) students significantly increased their ATS scores at post-test compared to pre-test. While there were no statistically meaningful distinctions among the three groups at time 2, the control group’s ATS scores did not exhibit a significant increase from pre-test to post-test (*p* = 0.774). It indicates that students in the intervention groups gained more knowledge about appropriate touch than before, albeit to a lesser extent.

Regarding the Inappropriate Touch Subscale, group 1 and group 2 both demonstrated a significant difference in scores between the pre-test and post-test (*p* < 0.001), while group 3 did not exhibit this difference. Furthermore, in multiple comparisons, the understanding of inappropriate touch was significantly higher in both group 1 and group 2 than in the control group (*p* = 0.003, *p* < 0.001). Overall, the participation in the Doll program significantly enhanced children’s grasp of CSA prevention knowledge, particularly regarding inappropriate touching. These outcomes align with prior studies that assessed CSA programs using the CKAQ-RIII [16, 45, 46].

Regarding Hypothesis 2, parental involvement appears to positively impact the understanding of inappropriate touching, as indicated by the significant difference in ITS scores (*p* = 0.016) between group 1 (child only) and group 2 (child and parent) at post-test. It is suggested that the inclusion of parents in the Doll program may have motivated children to explore more scenarios related to sexual abuse. Further research is necessary to delineate the specific positive roles played by parents in the Doll program.

Finally, the results reveal that while the control group showed no significant change in Appropriate Touch Scale scores, there was a notable increase in Inappropriate Touch Scale scores between pre-test and post-test (*p* < 0.001). This suggests a significant improvement in the control group’s awareness of inappropriate touching over time. One plausible explanation is that the pre-test acted as a motivator for some children to seek correct answers, leading to an enhanced understanding through discussions or increased attention to the subject matter. Another plausible explanation is that these children may have been exposed to related information through other curricular aspects. These findings are consistent with Tutty’s research, where children who had not participated in a prevention program still displayed noteworthy shifts in knowledge over a 5-month period, encompassing both aspects of touch appropriateness and inappropriateness [42]. Comparable outcomes were also noted in another study conducted by Tutty and in the implementation of the HOOC program for preventing CSA [41, 45].

### Limitations

First, it is crucial to highlight that our study solely involved the measurement of two data sets: the pre-test and post-test (5 weeks), demonstrating the Doll’s effectiveness in increasing children’s knowledge of sexual abuse prevention. However, it is noteworthy that our study did not delve into the long-term knowledge retention within the program, a dimension commonly scrutinized in other evaluation studies [17, 45, 51]. Hence, it is imperative to complement the Doll program with a follow-up evaluation.

Additionally, it is essential to refrain from assuming a direct link between improved knowledge, evaluated through the CKAQ-RIII, and subsequent behavioral changes leading to a reduction in the risk of CSA for participants [4, 46, 52, 53]. While there is a common assumption that increased knowledge and skills correspond to behavioral changes in real-life scenarios [54], evidence supporting the preventive impact of knowledge acquisition on sexual abuse is lacking [55]. The effectiveness of the Doll prevention program in reducing future instances of sexual abuse remains uncertain, necessitating additional research.

Furthermore, Sexual abuse prevention programs have faced scrutiny over the balance between program benefits and potential drawbacks, including inducing anxiety or impeding cooperation and trust in adults [56]. Hence, in addition to emphasizing the positive outcomes of CSA prevention programs, numerous studies have scrutinized potential negative effects. These evaluations encompass possible detrimental consequences such as anxiety, touch aversion, fear, mistrust of people, etc. [17, 47], [57–59], and found that the majority of children are not adversely affected by exposure to CSA prevention programs. Instead, children experienced an increase in knowledge of sexual abuse prevention [17, 58], a decrease in self-blame for sexual abuse [60], a rise in the disclosure of sexual abuse incidents [61], an improvement in parent-child communication [62], and no increase in sexual problems [63]. Our current study does not evaluate potential negative impacts of the Doll program. Subsequent studies will encompass a broader range of children to evaluate the program’s appropriateness.

Finally, while parents participated in our study, the Doll program’s main focus lies in fostering children’s awareness of sexual abuse prevention rather than delivering explicit instructions to parents. Critics argue that child-directed CSA programs place the prevention responsibility on victims [4], but it’s crucial to recognize their positive impact in enhancing children’s knowledge for self-protection. Simultaneously, there’s a moral duty to equip children with tools for effective action against sexual abuse [56]. However, Child-focused CSA programs are just one part of a CSA prevention. Preventing child sexual abuse requires not only educating and empowering children but also engaging parents, teachers, social workers and other stakeholders to participate in prevention programs [64–66]. In future studies, we need to iterate on the Doll program, involving other stakeholders such as parents and teachers, to ensure a more comprehensive impact.

### Conclusion

Despite these limitations, the findings carry significant implications. They suggest that the Doll program effectively enhances participants’ CSA knowledge, highlighting the efficacy of the e-educational toolkit in achieving substantial knowledge gains. This improvement could potentially lead to increased adoption of CSA prevention skills, ultimately contributing to lower rates of CSA—a crucial area for future research. The cost-effective implementation of the e-educational toolkit in the Doll program makes it particularly suitable for environments with limited educational resources, which provides valuable insights into preventing child sexual abuse in countries with conservative sexual education contexts and scarce resources, such as China. Additionally, the findings shed light on the impact of parental engagement on learning. Future research should focus on enhancing the Doll program, iterating the e-toolkit to involve a broader range of stakeholders, including parents and teachers, and providing training.

## References

[1] Barth J Bermetz L Heim E Trelle S Tonia T. The Current Prevalence of Child Sexual Abuse Worldwide: A Systematic Review and Meta-Analysis. Int J Public Health (2013) 58(3):469–83. 10.1007/s00038-012-0426-1 23178922

[2] Wurtele SK. Preventing Sexual Abuse of Children in the Twenty-First Century: Preparing for Challenges and Opportunities. J Child Sex Abuse (2009) 18(1):1–18. 10.1080/10538710802584650 19197612

[3] Stoltenborgh M van Ijzendoorn MH Euser EM Bakermans-Kranenburg MJ. A Global Perspective on Child Sexual Abuse: Meta-Analysis of Prevalence Around the World. Child Maltreat (2011) 16(2):79–101. 10.1177/1077559511403920 21511741

[4] Collin-Vézina D Daigneault I Hébert M. Lessons Learned From Child Sexual Abuse Research: Prevalence, Outcomes, and Preventive Strategies. Child Adolesc Psychiatry Ment Health (2013) 7(1):22. 10.1186/1753-2000-7-22 23866106 PMC3720272

[5] Okamura A Heras P Wong-Kerberg L. Sexual Abuse in Nine North American Cultures: Treatment and Prevention. In: Sexual Abuse in Nine North American Cultures: Treatment and Prevention. Thousand Oaks: SAGE Publications, Inc. (1995). p. 67–96. 10.4135/9781452243337

[6] Tang CS. Childhood Experience of Sexual Abuse Among Hong Kong Chinese College Students. Child Abuse Neglect (2002) 26(1):23–37. 10.1016/S0145-2134(01)00306-4 11860160

[7] Lyu Y Chow JC-C Hwang J-J. Exploring Public Attitudes of Child Abuse in Mainland China: A Sentiment Analysis of China’s Social Media Weibo. Child Youth Serv Rev (2020) 116:105250. 10.1016/j.childyouth.2020.105250

[8] Tang CS Lee YK. Knowledge on Sexual Abuse and Self-Protection Skills: A Study on Female Chinese Adolescents With Mild Mental Retardation. Child Abuse Neglect (1999) 23(3):269–79. 10.1016/S0145-2134(98)00124-0 10219945

[9] Lu M Barlow J Meinck F Walsh K Wu Y. School-Based Child Sexual Abuse Interventions: A Systematic Review and Meta-Analysis. Res Soc Work Pract (2023) 33(4):390–412. 10.1177/10497315221111393

[10] Chen Y-C Fortson BL Tseng K-W. Pilot Evaluation of a Sexual Abuse Prevention Program for Taiwanese Children. J Child Sex Abuse (2012) 21(6):621–45. 10.1080/10538712.2012.726699 23194138

[11] Jin Y Chen J Jiang Y Yu B. Evaluation of a Sexual Abuse Prevention Education Program for School-Age Children in China: A Comparison of Teachers and Parents as Instructors. Health Edu Res (2017) 32(4):364–73. 10.1093/her/cyx047 28854573

[12] Urbann K Bienstein P Kaul T. The Evidence-Based Sexual Abuse Prevention Program: Strong With Sam. J Deaf Stud Deaf Edu (2020) 25(4):421–9. 10.1093/deafed/enaa019 32696964

[13] Cecen-Erogul A Hasirci OK. The Effectiveness of Psycho-Educational School-Based Child Sexual Abuse Prevention Training Program on Turkish Elementary Students. Kuram Ve Uygulamada Egitim Bilimleri (2013) 13(2):725–729. Available: https://www.semanticscholar.org/paper/The-Effectiveness-of-Psycho-Educational-Child-Abuse-Cecen-Erogul-Hasirci/edd5686b4ec773d62c53c8dad3d5ddc6b15fd147 (Accessed September 13, 2023).

[14] Martin J Riazi H Firoozi A Nasiri M. A Sex Education Program for Teachers of Preschool Children: A Quasi-Experimental Study in Iran. BMC Public Health (2020) 20(1):692. 10.1186/s12889-020-08826-y 32410684 PMC7227303

[15] McElearney A Brennan-Wilson A Murphy C Stephenson P Bunting B. Cluster Randomised Controlled Trial of ‘Whole School’ Child Maltreatment Prevention Programme in Primary Schools in Northern Ireland: Study Protocol for Keeping Safe. BMC Public Health (2018) 18(1):590. 10.1186/s12889-018-5492-8 29724196 PMC5934867

[16] Jones C Scholes L Rolfe B Stieler-Hunt C. A Serious-Game for Child Sexual Abuse Prevention: An Evaluation of Orbit. Child Abuse Neglect (2020) 107:104569. 10.1016/j.chiabu.2020.104569 32535338

[17] Czerwinski F Finne E Alfes J Kolip P. Effectiveness of a School-Based Intervention to Prevent Child Sexual Abuse—Evaluation of the German IGEL Program. Child Abuse Neglect (2018) 86:109–22. 10.1016/j.chiabu.2018.08.023 30278285

[18] Song Y. The Sexuality Education and Attitudes of College Students in China. Health Edu (2015) 115(1):93–104. 10.1108/HE-01-2014-0002

[19] Huang S Cui C. Preventing Child Sexual Abuse Using Picture Books: The Effect of Book Character and Message Framing. J Child Sex Abuse (2020) 29(4):448–67. 10.1080/10538712.2020.1719449 32109197

[20] Wu Y Chen J Guo S. Knowledge, Attitudes, and Practice of Child Sexual Abuse Prevention Among Primary School Teachers: A Study in a City of Guangdong Province of China. J Child Sex Abuse (2021) 30(8):994–1005. 10.1080/10538712.2021.1985675 34635028

[21] Sherr L Mebrahtu H Mwaba K Nurova N Chetty AN Swartz A ‘Tipping the Balance’ – An Evaluation of COVID-19 Parenting Resources Developed and Adapted for Child Protection During Global Emergency Responses. Health Psychol Behav Med (2022) 10(1):676–94. 10.1080/21642850.2022.2104285 35957956 PMC9359164

[22] Sanders MR Divan G Singhal M Turner KMT Velleman R Michelson D Scaling Up Parenting Interventions Is Critical for Attaining the Sustainable Development Goals. Child Psychiatry Hum Dev (2022) 53(5):941–52. 10.1007/s10578-021-01171-0 33948778 PMC8096135

[23] Schafer M Lachman JM Gardner F Zinser P Calderon F Han Q Integrating Intimate Partner Violence Prevention Content Into a Digital Parenting Chatbot Intervention During COVID-19: Intervention Development and Remote Data Collection. BMC Public Health (2023) 23(1):1708. 10.1186/s12889-023-16649-w 37667352 PMC10476288

[24] Arnab S Brown K Clarke S Dunwell I Lim T Suttie N The Development Approach of a Pedagogically-Driven Serious Game to Support Relationship and Sex Education (RSE) Within a Classroom Setting. Comput Edu (2013) 69:15–30. 10.1016/j.compedu.2013.06.013

[25] Chan SL Jaafar A. Usage-Centered Design Approach in Design of Malaysia Sexuality Education (MSE) Courseware. In: Visual Informatics: Bridging Research and Practice. Berlin, Heidelberg: Springer (2009). p. 856–67. 10.1007/978-3-642-05036-7_81

[26] Jones CM Pozzebon K. Being Safety Smart: Social Issue Game for Child Protective Behaviour Training. In: Presented at the Proceedings of HCI 2010. Dundee, United kingdom: BCS Learning & Development (2010). Available: https://www.scienceopen.com/hosted-document?doi=10.14236/ewic/HCI2010.20 (Accessed May 10, 2023).

[27] Muller AR Roder M Fingerle M. Child Sexual Abuse Prevention Goes Online: Introducing ‘Cool and Safe’ and its Effects. Comput Edu (2014) 78:60–5. 10.1016/j.compedu.2014.04.023

[28] Pritha ST Tasnim R Kabir MA Amin S Das A. Smartphone Apps for Child Sexual Abuse Education: Gaps and Design Considerations. IJMLO (2023) 1(1):1. 10.1504/IJMLO.2023.10049097

[29] Endendijk JJ Tichelaar HK Deen M Deković M. Vil Du?! Incorporation of a Serious Game in Therapy for Sexually Abused Children and Adolescents. Child Adolesc Psychiatry Ment Health (2021) 15(1):25. 10.1186/s13034-021-00377-3 34034787 PMC8147575

[30] UNESCO. Revised Edition of the International Technical Guidance on Sexuality Education. Paris: UNESCO (2018). Available at: http://unesdoc.unesco.org/images/0026/002607/260770e.pdf (Accessed January 14, 2024).

[31] Ji Y Reiss MJ. Cherish Lives? Progress and Compromise in Sexuality Education Textbooks Produced in Contemporary China. Sex Edu (2022) 22(4):496–519. 10.1080/14681811.2021.1955670

[32] Liu W. Cherishing Life - Sexuality Education for Primary School Students (5 Grade I). Beijing: Beijing Normal University Publishing Group (2017).

[33] Sanders EB-N Stappers PJ. Co-Creation and the New Landscapes of Design. CoDesign (2008) 4(1):5–18. 10.1080/15710880701875068

[34] Ramos-Vega MC Palma-Morales VM Pérez-Marín D Moguerza JM. Stimulating Children’s Engagement With an Educational Serious Videogame Using Lean UX Co-Design. Entertainment Comput (2021) 38:100405. 10.1016/j.entcom.2021.100405

[35] Hussain S Sanders EB-N. Fusion of Horizons: Co-Designing With Cambodian Children Who Have Prosthetic Legs, Using Generative Design Tools. CoDesign (2012) 8(1):43–79. 10.1080/15710882.2011.637113

[36] Mencarini E Leonardi C Cappelletti A Giovanelli D De Angeli A Zancanaro M. Co-Designing Wearable Devices for Sports: The Case Study of Sport Climbing. Int J Human-Computer Stud (2019) 124:26–43. 10.1016/j.ijhcs.2018.10.005

[37] Babatsikos G. Parents’ Knowledge, Attitudes and Practices About Preventing Child Sexual Abuse: A Literature Review. Child Abuse Rev (2010) 19(2):107–29. 10.1002/car.1102

[38] Wurtele S Gillispie E Decker L Franklin C. A Comparison of Teachers vs. Parents as Instructors of a Personal Safety Program for Preschoolers. Child Abuse Neglect (1992) 16:127–37. 10.1016/0145-2134(92)90013-H 1544024

[39] Xu W Cheung M. Parental Engagement in Child Sexual Abuse Prevention Education in Hong Kong. Health Edu Res (2023) 82(4):376–89. 10.1177/00178969231159968

[40] Tutty LM. What Children Learn From Sexual Abuse Prevention Programs: Difficult Concepts and Developmental Issues. Res Soc Work Pract (2000) 10(3):275–300. 10.1177/104973150001000301

[41] Tutty LM. Child Sexual Abuse Prevention Programs: Evaluating Who Do You Tell. Child Abuse Neglect (1997) 21(9):869–81. 10.1016/S0145-2134(97)00048-3 9298264

[42] Tutty LM. The Ability of Elementary School Children to Learn Child Sexual Abuse Prevention Concepts. Child Abuse Neglect (1992) 16(3):369–84. 10.1016/0145-2134(92)90046-T 1617471

[43] Tutty L. The Revised Children’s Knowledge of Abuse Questionnaire: Development of a Measure of Children’s Understanding of Sexual Abuse Prevention Concepts. Soc Work Res (1995) 19:112–20. 10.1093/swr/19.2.112

[44] Daigneault I Hébert M McDuff P Frappier J-Y. Evaluation of a Sexual Abuse Prevention Workshop in a Multicultural, Impoverished Urban Area. J Child Sex Abuse (2012) 21(5):521–42. 10.1080/10538712.2012.703291 22994691

[45] Dunn M. The Learning of Sexual Abuse Prevention Concepts and the Reliability of the Ckaq-Riii in the South African Context. Social Work (2011) 47(2):155–75. 10.15270/47-2-133

[46] Gangos CJ Nega C Apergi F-S. Adaptation and Psychometric Evaluation of the Children’s Knowledge of Abuse Questionnaire (CKAQ-RIII) in Greek Elementary School Children. J Child Sex Abuse (2019) 28(2):222–39. 10.1080/10538712.2018.1538175 30403930

[47] Hébert M Lavoie F Piché C Poitras M. Proximate Effects of a Child Sexual Abuse Prevention Program in Elementary School Children. Child Abuse Neglect (2001) 25(4):505–22. 10.1016/S0145-2134(01)00223-X 11370723

[48] Weir KM. An Exploratory Study of Pre-Schooler’s Perceptions and Understanding of Concepts Taught in the ‘Feeling Special, Feeling Safe’ Sexual Abuse Prevention Program: A Thesis Presented in Partial Fulfillment of the Requirements for the Degree Master of Arts in Psychology at Massey University. New Zealand: Massey University (1999). Thesis, Available: https://mro.massey.ac.nz/handle/10179/6745 (Accessed September 13, 2023).

[49] del Campo Sánchez A López Sánchez F. Evaluation of School-Based Child Sexual Abuse Prevention Program. Psicothema (2006) 18:1–8. Spanish. 17296002

[50] Jin Y Chen J Yu B. Knowledge and Skills of Sexual Abuse Prevention: A Study on School-Aged Children in Beijing, China. J Child Sex Abuse (2016) 25(6):686–96. 10.1080/10538712.2016.1199079 27561123

[51] Hazzard A Webb C Kleemeier C Angert L Pohl J. Child Sexual Abuse Prevention: Evaluation and One-Year Follow-Up. Child Abuse Neglect (1991) 15(1):123–38. 10.1016/0145-2134(91)90097-W 2029665

[52] Zhang W Chen J Feng Y Li J Liu C Zhao X. Evaluation of a Sexual Abuse Prevention Education for Chinese Preschoolers. Res Soc Work Pract (2014) 24(4):428–36. 10.1177/1049731513510409

[53] Anderson LA Whiston SC. Sexual Assault Education Programs: A Meta-Analytic Examination of Their Effectiveness. Psychol Women Q (2005) 29(4):374–88. 10.1111/j.1471-6402.2005.00237.x

[54] Fryda CM Hulme PA. School-Based Childhood Sexual Abuse Prevention Programs: An Integrative Review. J Sch Nurs (2015) 31(3):167–82. 10.1177/1059840514544125 25092721

[55] Topping KJ Barron IG. School-Based Child Sexual Abuse Prevention Programs: A Review of Effectiveness. Rev Educ Res (2009) 79(1):431–63. 10.3102/0034654308325582

[56] Finkelhor D. The Prevention of Childhood Sexual Abuse. Future Child (2009) 19(2):169–94. 10.1353/foc.0.0035 19719027

[57] Casper R. Characteristics of Children Who Experience Positive or Negative Reactions to a Sexual Abuse Prevention Program. J Child Sex Abuse (1999) 7(4):97–112. 10.1300/J070v07n04_07

[58] Lee YK Tang CS. Evaluation of a Sexual Abuse Prevention Program for Female Chinese Adolescents With Mild Mental Retardation. Am J Ment Retard (1998) 103(2):105. 10.1352/0895-8017(1998)103<0105:EOASAP>2.0.CO;2 9779279

[59] Wurtele SK Miller‐Perrin CL. An Evaluation of Side Effects Associated With Participation in a Child Sexual Abuse Prevention Program. J Sch Health (1987) 57(6):228–31. 10.1111/j.1746-1561.1987.tb07838.x 3650596

[60] Finkelhor D Asdigian N Dziuba-Leatherman J. Victimization Prevention Programs for Children: A Follow-Up. Am J Public Health (1995) 85(12):1684–9. 10.2105/AJPH.85.12.1684 7503345 PMC1615746

[61] Walsh K Zwi K Woolfenden S Shlonsky A. School-Based Education Programmes for the Prevention of Child Sexual Abuse. Cochrane Database Syst Rev (2015) 2015(4):CD004380. 10.1002/14651858.CD004380.pub3 25876919 PMC9805791

[62] Finkelhor D Asdigian N Dziuba-Leatherman J. The Effectiveness of Victimization Prevention Instruction: An Evaluation of Children’s Responses to Actual Threats and Assaults. Child Abuse Neglect (1995) 19(2):141–53. 10.1016/0145-2134(94)00112-8 7780777

[63] Gibson LE Leitenberg H. Child Sexual Abuse Prevention Programs: Do They Decrease the Occurrence of Child Sexual Abuse? Child Abuse Neglect (2000) 24(9):1115–25. 10.1016/S0145-2134(00)00179-4 11057700

[64] Margolin L Wurtele SK Miller-Perrin CL. Preventing Child Sexual Abuse: Sharing the Responsibility. Fam Relations (1993) 42:109–10. 10.2307/584937

[65] Wurtele SK. Preventing the Sexual Exploitation of Minors in Youth-Serving Organizations. Child Youth Serv Rev (2012) 34(12):2442–53. 10.1016/j.childyouth.2012.09.009

[66] Robertson AL Harris DA Karstedt S. ‘It’s a Preventable Type of Harm’: Evidence-Based Strategies to Prevent Sexual Abuse in Schools. Child Abuse Neglect (2023) 145:106419. 10.1016/j.chiabu.2023.106419 37625366

